# Post-Acute Sequelae of SARS-CoV-2 Infection Among Adults Aged ≥18 Years — Long Beach, California, April 1–December 10, 2020

**DOI:** 10.15585/mmwr.mm7037a2

**Published:** 2021-09-17

**Authors:** Kyle Yomogida, Sophie Zhu, Francesca Rubino, Wilma Figueroa, Nora Balanji, Emily Holman

**Affiliations:** ^1^Long Beach Department of Health and Human Services, Long Beach, California; ^2^Graduate Group in Epidemiology, University of California, Davis, Davis, California.

Post-acute sequelae of COVID-19, also known as “long COVID,” is used to describe the long-term symptoms that might be experienced weeks to months after primary infection with SARS-CoV-2, the virus that causes COVID-19. Among persons with a previous COVID-19 diagnosis, estimates of the prevalence of sequelae range from 5% among nonhospitalized persons to 80% among hospitalized persons (*1*,*2*). Studies have analyzed the aftereffects of COVID-19, but few have assessed the demographic characteristics associated with long COVID (*3*,*4*). Health disparities resulting from pervasive structural and socioeconomic barriers in the U.S. health care system might contribute to differences in these effects and might continue to exacerbate existing inequities (*5*). To identify trends in post-acute sequelae, the Long Beach Department of Health and Human Services (LBDHHS) interviewed a random sample of 366 persons aged ≥18 years who received a positive SARS-CoV-2 test result during April 1–December 10, 2020. One third of the persons interviewed reported having at least one symptom 2 months after their positive test result, with higher odds of sequelae among persons aged 40–54 years, females, and those with preexisting conditions. Black or African American (Black) participants had higher odds of reporting dyspnea and myalgia/arthralgia compared with other racial/ethnic groups. Persons who were aged ≥40 years, female, Black, or who reported known preexisting conditions also reported higher numbers of distinct sequelae. As the number of recovered COVID-19 patients increases, monitoring the prevalence of post-acute sequelae among larger cohorts in diverse populations will be necessary to understand and manage this condition. Identification of groups disproportionately affected by post-acute COVID-19 sequelae can help develop efforts to prioritize preventions and treatment strategies, including vaccination of groups at higher risk for these long-term sequelae, and access to testing and care for post-acute sequelae.

Data were collected by LBDHHS (California Code of Regulations, Title 17) under the authority of the Long Beach City Health Officer during case investigation and follow-up. Among 28,594 Long Beach residents aged ≥18 years who received a positive SARS-CoV-2 reverse transcription–polymerase chain reaction (RT-PCR) test result during April 1–December 10, 2020, approximately 3% (791) were randomly selected for follow-up interviews. Persons with an intellectual or developmental disability or who had died were excluded from the study. During the first round of sampling, 400 persons with positive test results during April 1–August 26, 2020 were randomly selected. Because of the winter surge in cases, a subsequent round of sampling was conducted during August 27–December 10, 2020, in which 391 persons were selected. The second round of sampling maintained the same proportion of selected persons to all confirmed cases as the first round. Overall, 366 (46.3%) of the 791 selected persons agreed to be interviewed. Participants were interviewed during October 1, 2020–March 3, 2021 by telephone at least 2 months after the positive test result (median = 202 days; range = 78–368 days) using a standardized survey instrument. Interviews were conducted in English, Spanish, or Khmer. Questions were adapted from the 30-item California Reportable Disease Information Exchange COVID-19 case investigation questionnaire. Interviewers recorded questionnaire responses in Veoci, a secured virtual emergency operation center software application.

Multivariable logistic regression was used to assess associations between symptoms experienced 2 months after receiving a positive test result and participant characteristics (demographics and preexisting conditions) by calculating adjusted odds ratios (aORs) for having any sequalae and each of the most common sequelae. Multivariable Poisson regression was used to assess adjusted incidence rate ratios (aIRRs) for the number of reported symptoms.^†^ Racial and ethnic groups were combined, and persons identifying as both White and a racial minority group were categorized by their respective racial minority group. Because of limited availability of diagnostic and screening testing during the sampling phase, persons who experienced symptoms within 14 days before and 10 days after testing were classified as being symptomatic at the time of diagnosis.^§^ Disease severity at diagnosis was classified according to the National Institutes of Health groupings as mild, moderate, or severe (*6*). Because small sample sizes precluded analysis of specific preexisting conditions, a yes/no variable was used to indicate any preexisting condition.^¶^ Persons were given the opportunity to report symptoms not covered in the survey. Responses with related symptoms were combined for analysis.** All analyses were conducted in R (version 4.0.5; R Foundation); p-values <0.05 were considered statistically significant. Collection and analysis of human surveillance data fall under routine public health activities in the State of California and were exempt from Institutional Review Board review.

Among the 366 participants, the largest percentages were aged 25–39 years (144; 39%), female (207; 57%), and Hispanic/Latino (240; 66%) (Table 1). These were elevated relative to the general population because persons aged 25–39 years account for approximately 25% of the population, females for 50%, and Hispanic/Latino persons for 40%. Approximately one half (46%) of participants reported having a chronic preexisting condition before their COVID-19 diagnosis. Nineteen (5%) participants were hospitalized because of COVID-19. Participants reported an average of 5.26 symptoms (standard deviation [SD] = 3.82), and most (92.3%) experienced at least one symptom related to COVID-19 around the time of testing. Ageusia, parosmia/anosmia, myalgia/arthralgia, fatigue, and headache, were reported by 54.1%, 50.3%, 51.4%, 48.4%, and 46.4% of participants, respectively (Table 2). Two months after a positive SARS-CoV-2 test result, 128 (35.0%) participants reported an average of 1.30 (SD = 2.40) symptoms. Participants reported fatigue (16.9%), ageusia (12.8%), parosmia/anosmia (12.6%), dyspnea (12.8%), and myalgia/arthralgia (10.9%). The frequency of symptoms reported by persons 2 months after receiving a positive SARS-CoV-2 test result varied with the severity of illness at diagnosis; 55.5% reported severe/critical symptoms, 52.6% reported moderate symptoms, 29% reported mild symptoms, and 3.7% reported no symptoms (Supplementary Table 1, https://stacks.cdc.gov/view/cdc/109584). Nearly one third of participants (115; 31.4%) had symptoms at the time of interview; fatigue (50; 13.7%), dyspnea (38; 10.4%), and parosmia (35; 9.6%) were most frequently reported.

**TABLE 1:** Characteristics of participants interviewed regarding sequelae after recovery from COVID-19 (N = 366) — Long Beach, California, April 1–December 10, 2020

Characteristic	No. (%)
**Age group, yrs**	
18–24	42 (11.5)
25–39	144 (39.3)
40–54	111 (30.3)
55–64	39 (10.7)
≥65	30 (8.2)
**Sex**	
Female	207 (56.6)
Male	158 (43.2)
Genderqueer/Nonbinary	1 (0.3)
Race/Ethnicity*	
Hispanic or Latino	240 (65.6)
White	51 (13.9)
Black or African American	31 (8.5)
Asian	27 (7.4)
Native Hawaiian or Other Pacific Islander	5 (1.4)
American Indian	1 (0.3)
Unknown	11 (3.0)
**Hospitalized for COVID-19**	
Yes	19 (5.2)
No	347 (94.8)
**Chronic preexisting condition**	
Yes	170 (46.4)
No	196 (53.6)

* Racial and ethnic groups were combined, and persons identifying as both White and a racial minority group were categorized with their respective racial minority group.

**TABLE 2:** Frequency of symptoms reported by recovered COVID-19 patients on date of COVID-19 testing, 1 and 2 months after the positive test result, and on the interview date (N = 366) — Long Beach, California, April 1–December 10, 2020

	Time relative to positive test date, no. (%)			
Symptom	Test date*	1 month after	2 months after	Interview date^†^
**Any symptom**	**338 (92.3)**	**175 (47.8)**	**128 (35.0)**	**115 (31.4)**
**No. of symptoms, mean (SD)**	**5.26 (3.82)**	**2.01 (2.98)**	**1.30 (2.40)**	**0.99 (2.04)**
Ageusia	198 (54.1)	84 (23.0)	47 (12.8)	33 (9.0)
Myalgia or arthralgia	188 (51.4)	62 (16.9)	40 (10.9)	30 (8.2)
Parosmia or anosmia	184 (50.3)	80 (21.9)	46 (12.6)	35 (9.6)
Fatigue	177 (48.4)	88 (24.0)	62 (16.9)	50 (13.7)
Headache	170 (46.4)	56 (15.3)	39 (10.7)	28 (7.7)
Cough	152 (41.5)	51 (13.9)	30 (8.2)	20 (5.5)
Chills or shivers	136 (37.2)	31 (8.5)	20 (5.5)	11 (3.0)
Fever	135 (36.9)	33 (9.0)	18 (4.9)	11 (3.0)
Dyspnea	115 (31.4)	65 (17.8)	47 (12.8)	38 (10.4)
Sore throat	96 (26.2)	28 (7.7)	13 (3.6)	7 (1.9)
Rhinorrhea	76 (20.8)	22 (6.0)	11 (3.0)	7 (1.9)
Diarrhea	73 (29.9)	18 (4.9)	11 (3.0)	7 (1.9)
Brain fog	67 (18.3)	36 (9.8)	28 (7.7)	26 (7.1)
Other	69 (18.9)	34 (9.3)	35 (9.6)	39 (10.7)
Nausea	62 (16.9)	16 (4.4)	12 (3.3)	8 (2.2)
Subjective fever	40 (10.9)	10 (2.7)	7 (1.9)	5 (1.4)
Abdominal pain	34 (9.3)	10 (2.7)	3 (0.8)	1 (0.3)
Vomiting	32 (8.7)	5 (1.4)	3 (0.8)	3 (0.8)
Rash/Skin abnormality	8 (2.2)	5 (1.4)	4 (1.1)	4 (1.1)
Blood clots	3 (0.8)	1 (0.3)	1 (0.3)	0 (—)
Did not recall	1 (0.3)	1 (0.3)	2 (0.5)	0 (—)

**Abbreviations:** RT-PCR = reverse transcription–polymerase chain reaction; SD = standard deviation.

* Symptoms experienced within 14 days before and 10 days after date of first positive test result were classified as related to COVID-19 diagnosis.

^†^ Interview occurred a median of 202 days after collection of the specimen that yielded the RT-PCR result (range 78–368 days).

In the multivariate regression model, the odds of experiencing symptoms 2 months after a positive SARS-CoV-2 test result were significantly higher among females (aOR = 2.83), persons with at least one preexisting condition (aOR = 2.17), and those aged 40–54 years (versus 25–39 years) (aOR = 1.86) (Table 3). Analyses of the four most common symptoms experienced 2 months after a positive test result (fatigue, dyspnea, parosmia/ageusia, and myalgia/arthralgia) revealed similar findings in persons with at least one preexisting condition, females, and aged ≥40 years. (Supplementary Table 2, https://stacks.cdc.gov/view/cdc/109585). Females had higher adjusted odds of ageusia/parosmia/anosmia and fatigue than did males; whereas persons aged ≥40 years had higher adjusted odds of both, as well as myalgia/arthralgia, compared with persons aged 18–39 years. Persons with at least one preexisting condition had higher adjusted odds of all four of the most common symptoms compared with persons without preexisting conditions. Among Black persons compared with other racial/ethnic groups, the aORs of experiencing dyspnea and myalgia/arthralgia 2 months after testing were 2.52 and 3.67 times higher, respectively.

**TABLE 3:** Predictors associated with having any symptoms (logistic regression) and number of symptoms (quasi-Poisson regression) 2 months after COVID-19 diagnosis (N = 363)* — Long Beach, California, April 1–December 10, 2020

Characteristic	aOR/aIRR (95% CI)	P-value
**Multivariable logistic regression model of predictors (aOR)**		
Intercept^¶^	0.15 (0.09–0.27)	<0.001
**Sex**		
Female	2.83 (1.74–4.61)	<0.001
**At least one preexisting condition**	2.17 (1.35–3.5)	0.001
**Age group, yrs**		
18–24	0.66 (0.26–1.66)	0.38
25–39	Ref	—
40–54	1.86 (1.08–3.21)	0.03
55–64	1.57 (0.73–3.38)	0.24
≥65	1.47 (0.62–3.48)	0.38
**Multivariable quasi-Poisson model of predictors (aIRR)**		
Intercept^†^	0.41 (0.21–0.81)	0.01
**Preexisting conditions**	1.96 (1.32–2.91)	0.001
**Age group, yrs**		
18–24	0.73 (0.29–1.83)	0.50
25–39	Ref	—
≥40	1.73 (1.14–2.63)	0.01
**Race/Ethnicity**		
Asian	0.91 (0.41–2.04)	0.82
Black/African American	1.95 (1.02–3.73)	0.04
Hispanic/Latino	0.84 (0.49–1.43)	0.52
Grouped^§^	1.24 (0.51–3.01)	0.64
White	Ref	—
**Sex**		
Female	2.13 (1.40–3.25)	<0.001

**Abbreviations:** aIRR = adjusted incidence rate ratio; aOR = adjusted odds ratio; CI = confidence interval; Ref = referent group.

* Analysis excludes one person who identified as nonbinary and two persons with insufficient outcome data.

^†^ The intercept represents the expected mean aOR if a person identifies with all referent groups (e.g., in the quasi-Poisson model, the intercept represents the expected mean aOR of White males aged 25–39 years without preexisting conditions).

^§^ Includes American Indian persons, Alaska Native persons, Asian persons, Native Hawaiian or Other Pacific Islander persons, and persons who did not identify a race.

More symptoms were reported by females (aIRR = 2.13; 95% CI = 1.40–3.25), persons with preexisting conditions (aIRR = 1.96; 95% CI = 1.32–2.91), persons aged ≥40 years (aIRR = 1.73; 95% CI = 1.14–2.63), and Black persons (aIRR = 1.95; CI = 1.02–3.73) than by males, persons without preexisting conditions, persons aged 25–39 years, and non-Hispanic White persons (Table 3).

## Discussion

In this random sample of adults with a recent history of confirmed COVID-19, one third of participants reported post-acute sequelae 2 months after their SARS-CoV-2 positive test result, with higher odds among persons aged 40–54 years, females, and those with preexisting conditions. Persons aged ≥40 years, females, those with preexisting conditions, and Black persons also reported higher rates of post-acute sequelae. As the number of recovered COVID-19 patients increases, monitoring the prevalence of post-acute sequelae among larger cohorts in diverse populations is important because it can help develop efforts to prioritize prevention and treatment strategies for these populations.

These results are consistent with other published studies regarding age and female sex (*1*,*7*–*9*). Further, the associations between sequelae and preexisting conditions have also been reported by other investigators (*3*,*7*,*8*); however, few reports have assessed variations by race/ethnicity (*10*), which is important because of existing inequities that might lead to higher risk for SARS-CoV-2 exposure, lower access to care and testing, and differences in the prevalence of preexisting conditions in some racial and ethnic groups. The racial/ethnic variations observed in this study underscore the importance of continued efforts to reduce these inequities through prioritizing prevention and treatment strategies.

The findings in this report are subject to at least seven limitations. First, the results are based on a limited sample size, which resulted in large error estimates for some groups, especially for some racial/ethnic minority groups and for persons with certain preexisting conditions. Second, socioeconomic status, which was not assessed, might have resulted in unmeasured confounding away from the null. Third, it was not possible to attribute specific symptoms to SARS-CoV-2 infection, and the symptom assessment period (from 14 days before to 10 days after testing) might have included symptoms present before SARS-CoV-2 infection. Fourth, reported symptoms might vary over time because of recall bias. Fifth, severity of symptoms and associated functional impairments were not assessed. Sixth, participation bias might be present because those still experiencing symptoms might be more likely to respond, and hospitalization rates in the sample were relatively low. Finally, because the COVID-19 death rate was higher among all minority groups than among non-Hispanic White persons in Long Beach (LBDHHS, Communicable Disease Control Program, unpublished data, 2020), and because persons who were incapacitated or who had died were excluded from the analysis, the results might be biased by survivorship and access to testing.

Identifying disparities in post-acute COVID-19 sequelae can help guide the allocation of public health resources and improve health equity while groups recover from the long-term effects of the COVID-19 pandemic. Ensuring equitable access to care for persons recovering from long-term sequelae, particularly for those at a higher risk for sequelae, is important. In addition, preventive measures including physical distancing, consistent mask use, vaccination, and outreach can be prioritized or promoted for groups at an increased risk for experiencing long-term sequelae. Further research, including research over longer periods, is warranted to evaluate potential gaps in access to resources and care for persons with long-term sequelae across diverse populations and to better understand the role of the health determinants that drive these disparities.

**Summary**

**What is already known about this topic?**

The term “long COVID” is used to describe post-acute sequelae and long-term symptoms that can be experienced from weeks to months by persons recovering from COVID-19.

**What is added by this report?**

In a random sample of recovered COVID-19 patients in Long Beach, California, one third of participants reported post-acute sequelae 2 months after their positive test result, with higher rates reported among persons aged ≥40 years, females, persons with preexisting conditions, and Black persons.

**What are the implications for public health practice?**

Identification of populations disproportionately affected by COVID-19 and long COVID can help guide efforts to prioritize prevention and treatment.

## Footnotes

* These authors contributed equally to this report.

† Multivariable Poisson regression was used to assess adjusted incidence rate ratios (aIRRs) of the number of reported symptoms experienced 2 months after receipt of a positive SARS-CoV-2 test result across participant characteristics. Model assumptions for Poisson regression were assessed. Because of overdispersion, a quasi-Poisson link function was applied; all other assumptions were met. Forward stepwise selection was used to identify confounding variables using significance level of α = 0.05. Model selection was based upon minimizing the Akaike information criterion (an estimator of prediction error) as well as considering the public health significance of predictors with strong effect sizes.

§ These cutoffs were determined based upon previous knowledge that persons infected with SARS-CoV-2 might yield a positive RT-PCR result 2 days before symptom onset and most persons receive negative test results 10–12 days after symptom onset.

¶ Preexisting conditions measured in the questionnaire included diabetes, cardiovascular disease, hypertension, asthma, chronic lung disease, chronic kidney disease, chronic liver disease, stroke, neurologic or neurodevelopmental conditions, cancer, immunocompromising conditions, obesity, or history of smoking. Participants were allowed to report other preexisting conditions not directly assessed in the questionnaire. These included anxiety, depression, arthritis, allergies, hypothyroidism, chronic migraine headaches, fibromyalgia, and history of heart surgery.

** Responses related to joint pain (e.g., knee pain, joint pain, or bone aches) were categorized as arthralgia and grouped with myalgia for analysis. Responses related to alteration of sense of taste (ageusia) and changes in sense of smell (parosmia and anosmia) were grouped for analysis. Only symptoms reported by ≥40 participants were analyzed at the individual level.
