# *Erratum:* Vol. 70, No. 37

**DOI:** 10.15585/mmwr.mm7039a5

**Published:** 2021-10-01

In the report “Post-Acute Sequelae of SARS-CoV-2 Infection Among Adults Aged ≥18 Years — Long Beach, California, April 1–December 10, 2020,” on page 1276, in the first full paragraph the first sentence should have read, “In the **multivariable** regression model, the odds of experiencing symptoms 2 months after a positive SARS-CoV-2 test result were significantly higher among females (aOR = 2.83), persons with at least one preexisting condition (aOR = 2.17), and those aged 40–54 years (versus 25–39 years) (aOR = 1.86) (Table 3).”

